# A qualitative study of Filipina immigrants’ stress, distress and coping: the impact of their multiple, transnational roles as women

**DOI:** 10.1186/s12905-017-0429-4

**Published:** 2017-09-05

**Authors:** Melanie L. Straiton, Heloise Marie L. Ledesma, Tam T. Donnelly

**Affiliations:** 1 Division for mental and physical health, Norwegian Institute of Public Health, P.O. box 4404, 0403 Oslo, Norway; 2 Department of Child and Adolescent Psychiatry, Oslo University Hospital, Nydalen, P.O. Box 4956, 0424 Oslo, Norway; 3 Faculty of Nursing, Cumming School of Medicine, Community Health Sciences, University of Calgary, 2500 University Drive NW, Calgary, AB T2N 1N4 Canada

**Keywords:** Immigrant women’s mental health, Filipinas mental health, Post-colonial feminist perspective, Coping, Labour migration, Transnational marriages

## Abstract

**Background:**

Migration is associated with a number of stress factors which can affect mental health. Ethnicity, gender and socioeconomic status can intertwine with and influence the process of migration and mental health. Philippine migration to Europe has increased in recent years and has become more feminised. Knowing more about the factors that influence immigrants’ mental health and coping can help aid health care delivery and policy planning. The purpose of this qualitative study was to explore the contextual factors that influence the mental health of Filipinas living in Norway and their coping strategies.

**Method:**

Individual in-depth interviews were conducted with fourteen Filipinas 24–49 years, living in Norway. The analysis was informed by the post-colonial feminist perspective in order to examine the process by which gender, ethnicity and socioeconomic status interact with contextual factors in these women’s lives and influence their wellbeing.

**Results:**

Data analysis revealed that all informants experienced some level of stress or distress. Two main factors: *Sense of belonging* and *Securing a future* contributed to the women’s level of distress associated with living abroad as an immigrant woman. Distress was heighted by the women’s multiple, transnational roles they occupied; roles as workers, breadwinners, daughters, wives and mothers. None of the women had sought professional help for their distress. Religion and informal support from friends and family appear to help these women cope with many of the challenges they face as immigrant women living and working abroad.

**Conclusions:**

Filipinas face a number of challenges related to their status as immigrant women and the juggling of their transnational lives. Understanding the context of these women’s lives may aid the identification of mental health problems. Although the women show resilience and appear to cope successfully, some may benefit from professional help.

## Background

Migration is associated with a number of stress factors which can affect mental health [1]. There is therefore the concern that immigrants are at greater risk of mental health problems than the native population [2] and simultaneously face barriers to accessing appropriate mental health care [3]. Yet, the level and type of, and response to, migratory stress varies with, among other factors, individual characteristics, the circumstances of migration and the welcoming in the new country [1]. An individual’s ethnicity, gender and socioeconomic status also intertwine with and influence the process of migration, adaptation to the host society [4] and thus, mental health.

In Norway, immigrants now make up 14% of the population [5]. Norwegian health care providers and policymakers face new challenges in order to meet the needs of the growing immigrant population. In recent years, there has been an increase in women from the Philippines migrating to Norway for work and marriage. Despite Filipinas being the largest group of non-EU immigrant women in Norway [6], little is known about their mental health. This study focuses on Filipinas living in Norway and explores the stress and distress experiences associated with being an immigrant woman and how these women cope with their difficulties.

The Philippines is one of the biggest export countries of labour, with over 10 million Filipinos working or living abroad [7]. Migration is encouraged by the government, since the sending home of remittances helps to support the country’s economy. In 2014, personal remittances from overseas Filipino workers accounted for 8.5% of the gross domestic product [8]. Early labour migration consisted predominately of men but due to the changing global labour economy, women now outnumber men [9]. In Norway, 80% of immigrants from the Philippines are women [6]. Gender ideologies, including the traditional division of labour, shape migration patterns. Many Filipinas moving overseas help meet the shortage of skilled nurses as well as the demand for unskilled, low paid domestic work in high income countries [9, 10]. Increasingly, Filipinas also become the wives of men from high income countries, including Norway [11].

Filipina immigrants are often considered resourceful, with many having higher education [10, 12] and strong social support networks [13]. Nonetheless, they are sometimes considered at risk of violence, isolation, poverty and exploitation [13–15]. Reports of poor working conditions and abuse among foreign domestic workers for instance are not uncommon [16]. Further, more than 40% of labour Filipino migrants in the USA report high levels of workplace discrimination [17]. Such factors are also associated with poorer mental health, yet mental health among Filipinas has rarely been addressed in the literature. An Australian study found that Filipina immigrants more often report mental distress than Australian women [18]. In addition, financial stressors, relationship adjustments and social isolation were important factors in influencing the mental health of Filipinas married to Australians [19]. This highlights the importance of considering the social context of women’s lives and their migratory pathways in relation to mental health.

A recent Norwegian study found that Filipinas are less likely to consult with a general practitioner (GP) for mental health problems than Norwegian women [20]. Further, among those who did consult with a GP, they were less likely than Norwegian women to use psychotropic medicine or to engage in conversational therapy with the doctor. It is not known if Filipinas’ underrepresentation is because they have better mental health, if their distress is not recognised by a professional, if they experience barriers to care or if they seek help elsewhere. An improved understanding of the factors that influence Filipinas’ mental health can help the identification of mental distress and effective coping, with implications for the prevention and treatment of mental health problems. The purpose of this exploratory qualitative study is to illuminate the contextual factors that influence immigrant Filipinas’ mental health and their coping strategies. Ethical approval was obtained from the Regional Committee for Medical and Health Research Ethics, West Norway.

## Method

### Interview procedure

Using purposive and snowballing [21] sampling techniques, potential informants were identified and recruited through key personal contacts in the Filipino community. Selection criteria included being over the age of 18 and having lived in Norway one to 10 years. Women meeting the selection criteria were contacted via telephone or e-mail, informed about the study and asked to consider participating. They were sent written information in English, Norwegian or Filipino, about the goals of the study, what participation involved, rights and ethical considerations. A suitable time and place for the interview was then arranged. Informants could request a Filipino interpreter. Informants were also asked to tell their contacts about the study and those who wished to participate contacted the researcher. Consent for voluntary participation was obtained from each informant. Anonymity and confidentiality was assured.

Informants first completed a short questionnaire with background information and the ten-item Hopkins symptoms checklist (HSCL-10), a reliable measure of psychological distress [22]. This is a shortened version of the HSCL-25 which has been applied transnationally [23, 24]. Semi-structured interviews were then carried out; open-ended questions related to topics such as living in Norway, family background, emotional difficulties, perceptions of physical and mental health and experiences in consulting with general practitioners in Norway. The interview schedule was flexible depending on the dynamics of the discussion between the interviewee and interviewer.

Data collection continued until data saturation was reached and relatively little new information was obtained. Fourteen women were interviewed, all by the first author, a native English speaker, fluent in Norwegian. Thirteen interviews were conducted in English and one in Norwegian. None of the women required an interpreter. Interviews were audio-recorded and lasted on average 60 min (range 25–100 min). They were transcribed verbatim, yielding 277 A4 pages.

### Sample

The women had lived in Norway between one and 6 years (mean = 3.1 years) and were aged 24 to 49 years (mean = 33.7 years). Seven were married at the time of interview, ten had college or university level education and all but one were employed and/or studying. Six women were mothers (mean = 1.83 children), three of whom had children in the Philippines. Four of the women had initially moved to Norway through the au pair scheme (a cultural exchange with a host family in return for domestic work and childcare ([14])), four to find skilled work, four had a Norwegian spouse/partner and two for other reasons. Five of the women scored above 1.85 on the HSCL-10, suggesting they were experiencing psychological distress around the time of the interview.

### Analysis

The post-colonial feminist perspective was used to analyse the interview data. We used this perspective as a lens to give analytic depth to the many sided factors that affect Filipina immigrants living in Norway. The postcolonial feminist perspective can be applied to examine how ethnicity, gender, and socioeconomic position influence the social, cultural, political, historical and economic factors that shape the lives of marginalised women [25]. This method of critical enquiry allows us to challenge the assumptions of dominant society and helps to expose power imbalances that marginalise immigrant women. Additionally, the post-colonial feminist perspective notes that assumptions made about women are not universal. Rather, women around the world have different histories, cultures, ethnicities and socioeconomic circumstances. These factors influence women’s experiences and struggles [26].

We critically examine how contextual factors interact with ethnicity, gender and socioeconomic status [27] to influence the mental health of Filipinas and their coping strategies. We consider how gender shapes these women’s experiences and how their status as immigrants and the structural barriers they may encounter influence their circumstances and their mental health. We aim to understand these women’s stories through gender, cultural and political structures. We go beyond describing and categorising immigrant women’s mental health experiences and instead illuminate the socio-political and cultural conditions that influence these mental health experiences, and thereby giving immigrant women a voice. In this way, mental health issues and ways of coping are identified and addressed from the perspectives of women. We hope to generate an accurate account of the women’s lives and their mental health and coping strategies.

Transcripts were read and reread to allow familiarity with the data. Notes and initial impressions were made about each case. The data were then coded case by case before comparing and contrasting categories across the data. Special attention was paid to aspects of mental health, stress, sadness, daily hassles and coping in the context of what was known about these women’s lives. NVivo was used to assist in the coding and categorising of the transcripts. All citations used in the findings are anonymised to protect the women’s identities.

## Results

Five of the women reported having suffered from depression or anxiety at some stage in their life, with one having experienced this for the first time in Norway. However, some level of stress or distress associated with living abroad as an immigrant woman was reported by almost all of the informants. They described a mix of symptoms such as crying, low mood, confusion, anxiousness, rumination, feelings of helplessness, guilt, sadness and frustration, concentration difficulties, social withdrawal, restlessness and weakness, changes in appetite and sleeping difficulties. Somatic symptoms such as stomach problems, dizziness, headaches and muscular pain were also mentioned by some women.

Data analysis indicated two main factors relating to the experience of being an immigrant which contributed to stress or distress at a general level: *Sense of belonging* and *Securing a future.* These are described below. We then move on to show how these experiences are intertwined with the (often multiple) roles as breadwinners, workers, daughters, wives and mothers that these women inhabit. Finally, we show how the women’s strength and resilience appears to help many cope successfully with the challenges they face.

It should be noted first, that although the informants reported experiencing stress or distress at some point in Norway, they also conveyed a number of positive mental health experiences. The young, unmarried women, in particular, expressed a strong sense of empowerment associated with their move. They felt more independent and acknowledged a personal growth through their experiences “*I’m growing and making myself strong, facing different difficulties, different challenges*.” One woman who had suffered from a mental health problem off and on throughout her life felt moving abroad made her realise her strength and coping ability. By being pushed to experience a new way of life in a different culture and society, she felt more self-reliant:

*“I found out, recently that I am able to adjust. With wherever I am, I am able to adjust, and back in the Philippines I wasn’t able to do that… I’m living with some people and… that’s new for me and I find that I kind of like it as well. Because… it shows that I’m kind of independent now*.”

### Sense of belonging

The women, far away from their home country, most commonly described experiencing feelings of loneliness and homesickness, which could affect both physical and mental health: *“At first it’s veeery, very difficult. I keep on crying, my blood pressure will be [high]… and now my health is not good, because… of what I feel*.”

Adjusting to the colder climate was challenging for many of the women and the darkness in winter was particularly associated with homesickness. The women commonly spoke of language barriers and felt that learning Norwegian was “*the most important*” aspect for understanding and functioning in Norwegian society. However, even those who were proficient in Norwegian still felt a sense of alienation:

*“even though we have learned it…for how many years– I still have that, you know, alien feeling when some of them… look how Norwegians talk and then… they use some expressions …that is really funny, I wish that I could have understood it 100%*.”

Egalitarianism, a basic principle in Norwegian society, which values equality of all people [28] was brought up on occasion. For example, the women suggested that they did not feel that they were treated differently for doing low-skilled work. Yet, on other occasions, informants felt others sometime made assumptions about them based on their ethnicity and gender, causing them feel like an outsider of a lower social status.

*“I am a foreigner ... one can always see that: “Okay, maybe she is married to a Norwegian, or she is an au pair”, because that is always a thing here. When you have brown skin and black hair, it’s either you are married to a Norwegian, or you are an au pair, yeah.”*

The most salient factor impacting on the women’s sense of loneliness and not belonging, however, was the absence of their family. The women described the contrast in having been constantly surrounded by extended family all their lives to suddenly being in a strange and quiet environment, sometimes living, or at least spending more time, on their own. Even the women who had a Norwegian spouse and/or child in Norway felt a pronounced sense of loneliness without their kinship nearby: *“I don’t like [it here] first time I see. I don‘t like this, I don’t have family here. It’s so boring, it’s so -It’s only me, I don‘t have family, I don’t know what I should do.”*

### Securing a future

While it was not uncommon for the women to highlight that they were happy in Norway because they felt safe “*that’s what I like here, the security. The security I have, economically… physically, mentally here”*, reaching this stage was not an easy process, particularly for those who moved to find skilled work or who came as au pairs. Most informants had intentions of staying in Norway long-term but were faced with structural barriers related to visa restrictions that they need to overcome. These barriers, coupled with daily stresses and worries about their family at home, meant their uncertain residence status could affect both their physical and mental health.

“*I’ve been to the doctor, because I had this check-up, because sometimes I had this spotting. And then I understand, because probably it’s… stress, [I] think about Philippines, think about my visa here. Think about the exam for the Norwegian language, so that was the time period I was thinking about the bad things*.”

To obtain a skilled-workers visa, one has to be offered a job related to his/her education, of at least 30 h per week [29]. Visas are renewable every year but after 3 years, Filipinos can apply for permanent residency. The initial period of finding work was particularly stressful for women who came to Norway for this reason as they were often only offered part-time or temporary contracts.

For those who came through the au pair scheme, the situation could also be very uncertain, given the temporary nature of the work. According to legislation, people under 30 years can take part in a cultural exchange by living with a host family in exchange for help with light household chores and responsibilities such as childcare, cooking and cleaning [14]. The cultural exchange can be for up to 2 years and, as of 2012, is only for unmarried, childless individuals. After 2 years as an au-pair, women have no right to claim continued residence, unless they obtain skilled work, enrol in further study or marry a resident. Indeed, the au-pair scheme may often be a preferred option for some educated women to come to Norway [30]. They use this time to learn the language, culture and exchange their qualifications, while earning a small allowance and beginning the ground work for finding more relevant employment [30]: *“My goal was…to apply for work. Because I had my authorisation as a health worker and I have my Norwegian exam and then I can actually go out and find a job”.* Others however, may have no option but seek similar au-pair opportunities in other countries, continuing the cycle of insecurity and distress:

*“Especially when I have this depression…I can’t concentrate - everything, because I was like thinking too much about this. Because I am going to, I have two months left, and I need to find a family and everything… I mean this past month I am really stressed, because of this paper.”*

The women who moved to be with a spouse/partner (family reunification) did not feel exempt from insecurities about the future. One woman, for instance, who lost her husband, her biggest source of support, worried about her prospects:

*“when he died, my husband, I was really afraid ... what will happen to me. Because then I wasn’t even finished studying. So I thought, how? Also I am just a foreigner. And at that time I wasn’t good at speaking Norwegian.”*

### Role as worker

Many of the women were de-skilled upon coming to Norway; working as au pairs, waitresses or cleaners regardless of their migration pathway, despite having college or university level education. Nursing, a highly feminised profession, is a commonly chosen career for Filipinas because of the opportunity for overseas employment [10]. However, due to differing educational requirements between Norway and the Philippines, nurses often end up in lower-skilled positions with lower pay, such as care assistants, or in less preferred care work with unsociable hours [31]. This left the women feeling devalued and disillusioned when faced with the uphill struggle of further study in order to qualify as a nurse in Norway, particularly when the requirements were constantly updated:

*“When it comes to career, it’s the bureaucratic system that makes everything difficult ... sometimes I feel hopeless. It’s very frustrating you know, making use of your money and time for anything, for everything you have done.… so for me, right now I’m not really 100% satisfied because of… the hard work I’ve been through and nothing* *after how many years…. I have that feeling that I’m not yet done. it’s endless exams… So, because of changes and the… permanent system or check list for us to be… accepted here as nurses… is really hard…It’s not permanent. It’s not consistent.”*

As mentioned above, the au pair scheme was sometimes a stepping stone to more permanent employment for women and the host family could be a good resource when planning a future in Norway: *“the family was very… engaged and helpful, and supportive… in my dreams and in my plans”.* However, the role of an au-pair is open to interpretation and these women have the potential to be exploited “*some Filipinas were not lucky enough to have families who would really-... the word au pair, you are a worker, you are not a member of the family.”* Although revised regulations have increased the protection for au-pairs, it is not clear how this is controlled [14]. Host families of au-pairs are for instance obligated to pay for Norwegian tuition [32] but one informant indicated that she was denied this: “*I didn’t go to school because the second host family, they didn’t give me this. They were like okay, okay but nothing!*” This served to maintain the language barrier and limited her future opportunities in Norway, thus increasing the stress about her uncertain future. Although various regulating bodies can be contacted in these situations, this is unlikely to be an option for most of these women. Being young, female and in a foreign country, as well as financially reliant on the family, living in their home and having temporary immigration status leaves the women in vulnerable positions.

### Role as breadwinner

Almost all informants reported sending remittances home to family members; to support their children, parents, siblings or nieces and nephews. Given the importance of the family in Filipino culture [33], this was a natural thing for the women to do:

*‘so when it comes to financial support… I don’t take it as a responsibility. Literally – it’s not. But for me to get an amount like this – because I know I’m earning more than what they earn, it’s like… it’s a grateful thing’.*

While some were just providing a little extra to their families when they could, others were the main breadwinner, supporting their family with day to day living, housing, health services and education. The level of responsibility could be stressful for the women when they were unable to meet their families’ expectations. Feeling that their family members back home saw them as a never ending source of money, they sometimes struggled to make them understand differences in the cost of living and the financial obligations they had in Norway. This resulted in guilt and a sense of powerlessness:

*“And then I said to my mother that I only earn this money, so you don’t have to think about your sisters, your brothers’ family, because….we cannot afford to pay - to feed them all. Think of yourself, this is enough, this is what we have, and whatever extra you have, that’s the one that you have to give to them. Anyway I’m thinking about them, you know, but I cannot, I am not a God who can afford to feed them all.”*

### Role as a daughter

To some extent, women’s migration can be used as a strategy for resisting gender normative roles by giving them financial independence and more autonomy over their own lives [34].

*“In Norway so I get a sense of… a new outlook of how a [young adult]… would be like in the society. So I get my independence, I earn a lot; I can travel without the consent of my family. So it’s very new and a very noble experience*.”

However, the normative role as the dutiful daughter or altruistic mother means that women would sacrifice their own needs in order to send larger remittances home [35]. Indeed, this was evidenced by some informants; they took on multiple jobs to the point of exhaustion, engaged in studying in order to improve future earnings, forwent trips home to visit family and even delayed motherhood.

*“It’s not easy. So that’s also why it’s nice also that I don’t have a child yet….Sometimes I wish…when I saw kids, but I say, yeah it’s not yet time. I have to have work first… Maybe [then] it would be easier to support both”.*

In the Philippines, the sense of family obligation is particularly strong for the eldest daughter [36]. There is often the expectation that she will take on a caring role for younger siblings and aging family members. In families where parents invest in the eldest daughter’s education and/or help them go abroad, she is expected to contribute financially to the household, often supporting younger siblings. By putting the family’s needs before her own, she fulfils the role as a virtuous and moral daughter or sister.

The woman’s role as a nurturer is strongly embedded, which sometimes leads to feelings of guilt for being unable to provide direct care for aging family members back home [37]. Some rationalised that providing financially was more important “*whether we like it or not, practically speaking, I think the financial aspect weighs heavier.*” In this way, money becomes a way of showing care to loved ones [38]. However, it could not always compensate, as indicated by this woman working in health care:

*“I always tell myself that, because I know I will be living here, because I am working here, the love and the care that I give to my patients – are the love and care that I can never give to my grandmother and my mother. Because they will be there in the Philippines, and I am here, working… I can send money, I can call, but I mean the mere presence of being there…. It is very different when you touch a person… And I can do that with my patients. I can even wipe the saliva that drooling out. I can even clean, I can even bath them, I can even feed them. But, I can’t do that to my mamma, when she will grow old. I cannot do that to my ailing grandfather or grandmother.”*

Through engaging in paid emotional labour [39] this informant’s remittances had more meaning. In this way she could fulfil both her role as a nurturing woman and a provider, resolving some of her guilt.

### Role as a wife/partner

The role of breadwinner could be even more complex for some of the married women, who noted that the cultural differences in attitudes between Filipinos and Norwegians had potential for conflict: “*[Norwegians are] more independent. Your mums’ money, is your mums’ money, my money is my money, like that… but in the Philippines is share, share, share.”*

The women indicated that their spouses/partners were generally supportive of them sending remittances home and often helped out. However, there were times when the women felt torn between their family back home and the needs of their own household in Norway, which caused them distress:

*“Especially when you cannot send money or there is a problem there. So you think a lot. So it’s not easy. That’s the time I have problem sleeping… that’s the most difficult, you cannot do anything and... if I need to send money there, I cannot always ask my husband for money, because he has lot of bills, yeah. So I was in the in-between. I have to think about our own problem and then think of the family. Sometimes it’s really difficult – what should I do? … what should I prioritize to help my husband or, yeah… It’s not easy”.*

Feelings of disempowerment were also often described by the women with partners/spouses. The women who migrated to be with a partner/spouse were faced with a new and different culture, language and rules and regulations, placing them in a position of reliance on their husbands, emotionally, socially and economically. Although facing less pressure to secure their future through skilled work, to be granted permanent residence, women must now have lived in Norway with their husbands for 5 years [40]. Although the informants in this study described their husbands/partners as being kind, understanding and supportive, this legislation puts immigrant women at risk of being exploited or abused by their husbands [15]. Indeed, there was a worry that one could ‘*be thrown back to the Philippines again’* if the husband chose to end their relationship. In the very least, the power imbalance can serve to maintain ideologies of women as good and obedient housewives [41].

Further, due to visa restrictions on working prior to approval of a family reunification residence permit, women are often initially financially dependent on their partner. This also contributes to a power imbalance. Lacking their own income, women in this stage felt that they had lost some of their independence which affected their self-esteem:

*“Maybe I am just sad… and I told my husband that. I said: “I’d like my own income”. Because for now it's been long time that he’s giving me - even a simple [thing]...your own make-up or something. Of course you like to buy things on your own… I am sad because for now I cannot do anything….But I’m really, I want to have a job just for my own, my own...For yourself - you feel independent and confident.”*

In contrast, women who were married to Filipino men did not experience this economic dependence. They had been the first to move to Norway, with (or plans for) their husbands to follow through family reunification. Although this gives the women more autonomy and economic freedom, it can place a high amount of pressure on the women to successfully establish themselves with a skilled job, good income and a place to live. This process can take several years and the separation from their spouse can be a difficult period emotionally, increasing their sense of loneliness:

*“It was quite difficult for me to live alone… but then when he came here it was a relief, like everything opened for me… because I was always dependent emotionally with my husband. So it’s different when you talk to him through Skype or viber. It’s different when you have him with you*.”

### Role as a mother

The women who had children in the Philippines experienced extra emotional strain due to the separation:

*“it’s very hard ... the first time I arrived here I was crying every now and then ... you know, you are leaving your kids– I feel like dying. And then, as a mother, even now, I’m used to it, but still you cannot just “oh”. Sometimes … it [makes me] feel so weak, depressed also. Sometimes I really miss my kids – how I wish I could ... tie their hairs or something. Put them in bed, but I’m here so, yeah, it’s very sad”.*

Transnational families are not uncommon around the world. However, in addition to the sadness the women experience being away from their children, they can also feel somewhat judged by their decision to move abroad in order to provide financially for their children: “*some people told me that I was so tough, because here leaving kids is abandoning, but in my culture it’s sacrifices, it means a lot for us as a mother.”* This is not the same for fathers; the absence of fathers due to breadwinning overseas is more acceptable for both the affected children and at the societal level [42]. The ideology of women as nurturers again increases the guilt that the women experience as they try to mother their children from a distance. Although migration is often construed as a personal choice, leaving their children is not. These women are coerced into doing so as a result of their socioeconomic circumstances and the global economy [39]*: ‘how can I produce money? Even I work a 24 hours, 24/7 in the Philippines, [it’s] not enough’*.

While these breadwinning, financially independent mothers experienced emotional difficulties due to the separation from their children, the women who were mothers to young children in Norway experienced social isolation due to being primary caregivers and were financially and socially dependent on their husbands. Social isolation has been attributed to depressive symptoms among immigrant mothers [43]. Feelings of being restricted due to a husband’s demanding working hours, could impact the women’s mood considerably:

*“I cannot do anything now, before I can do this, do that, but now – oh no, I am stuck here in the house with my baby… It is really a different ambience for me, and I’m really not getting used to it.”*

### Resilience and coping

Despite being faced with a number of competing pressures due to the multiple roles these women occupy, together with adapting to a new life, these women showed resilience and strength. They felt they were able to adapt to the culture, climate and being away from family through time. They normalized both their loneliness and financial pressures from family: *‘I think it is a common one’* and showed a positive mind frame *“I adjust… I am here now so I have to go out and look for work, and do things, which is good, you know… Don’t look for the negative side.”* While mothers felt guilty and sad for not being with their children, at the same time, it was precisely their children who gave them the strength to continue: *‘sometimes it gives me like- okay, there’s no time for giving up, I’m tough, I’m strong. So okay, I don’t care. I need this for my kids’.*

The women engaged in various coping strategies, both emotion-focused such as crying or writing down problems to vent their feelings, and problem-focused ones such as changing their situation when feeling low:

*“If I can feel something attacking, I have to go out. I don’t want something get inside to my mind, because probably making me then [feel depressed]. So I have to go out and run. Running, walking or with some friends. Go downtown and eat”*.

Two main sources of support were emphasised by the women as important for coping with stress and distress; informal support and religion. None of the women had sought care from a health professional for their stress or distress, even amongst those who reported having experienced a mental health problem. Just as the women had transnational roles, many of them also had transnational support. This helped them to *maintain family ties* and to *re-create a sense of belonging*.

### Maintaining family ties

Frequent contact with family at home was important for reducing homesickness. Video calls and social media were helpful for maintaining close ties and giving a sense of being an active part of a loved one’s lives despite the distance. This was particularly important for the mothers as they felt it gave them a level of intimacy with their children. The belief that their children understood the reasons for their absence, also appeared to help the mothers resolve some of their guilt [42],:

*“Even though I am… so far from them. I keep on … looking at them. Contact with them. Constantly. Follow up what they doing…How do they feel today, how do they feel that I am not – I am far away from them… And they say that’s okay. Sometimes they felt so lonely, but it is okay. Because they know that my purpose is for them also. It’s important that they understand my situation also...So not they think that oh, I just go and have some fun here.”*

Many women had travelled back to the Philippines to visit family or had had visits from family members. Some also had, or had had family members living in Norway; siblings, aunts or cousins. This generally acted as a buffer against the loneliness they otherwise might have felt: *“A lot of people have been saying they get lonely from time to time,—I guess I get that way too, but… I have family here, though….So… I think I’m okay*”.

The strong sense of shared identity in Filipino culture [33] means that responsibilities and concerns are divided and therefore the burden of personal problems are shared. Despite the distance, families back home remained the main source of support for some – both emotionally and practically. For instance family members assumed joint responsibility for looking after children or were confidants and advice givers: *“Even now, sometimes I have a problem here, I talk to my family [back home] and it’s really a big help. Because… some private things you cannot talk to your friends…. I talk more to my, especially to my mom and my sister, all the sisters.”*

However, this was not the case for all women: “*Because most of us Filipino, we are thinking first what will my loved ones will think. I want to solve it first to myself, before telling to them. Because I am sure they are very affected”.* The collective concern for others and fears of destroying the hope that the breadwinning women represented for their family made it difficult for some to confide in their family: *“I am also one of the wings of my family, you don’t want them to see me flying down… I want them to see me…soaring high every time*.”

### Re-creating a sense of belonging

The establishment of a close-knit network of other Filipinos in Norway was not only one of the biggest protective factors for these women’s loneliness but also for adjustment. The openness of the Filipino community meant that friendships were often quickly built out of similarity and understanding for each other’s situations. Being able to speak their own language, cook Filipino food and eat together gave them a sense of familiarity and belonging that was otherwise lacking for them.

*“We have a group here… and that is the first time that I really feel more comfortable, because I have, I found my society…Filipino society here. I meet a lot of Filipina and we do the cultural evening … it’s like you don’t miss your country because you have your own country here”.*

Being part of such a group, formal or informal, strengthens ethnic identification, which is associated with lower levels of depressive symptoms [44] and provides strong emotional, practical and social support. The women turned to each other for advice when job hunting, when experiencing stress or difficulties in Norway or for comfort when they had family worries. They socialised, laughed and cried together and confided in each other. Through re-creating a sense of belongingness, the friend network became a substitute family for many.

### Religion

Majority of the women identified themselves as Catholic. Many attended church which functioned as a social arena to make new friends, a place to discuss problems and to feel a mutual sense of support:

*“Because Sunday is church day, and at* [the local church]*you can see a lot of Filipinos there during Sundays, having - going to the mass. If you’re new in a place you just: “oh, I’m a Filipino, are you?” So in that way… I met a lot of friends.”*

The social role of the church was important in times of need, for instance, when there were natural disasters at home. Additionally, some women indicated seeking advice from clergy. Spirituality however, seemed to be of greater significance to the informants in this study, whether or not they attended church. Their beliefs provided them with comfort and uplifting thoughts and they felt strong to enough to face difficult situations:

*“I guess that my religion has helped me a lot …. Giving me hope, and faith, really helped me a lot. That’s why I don’t really feel a feeling of hopelessness, because I know as long as you live, you have hope. And there’s a way always.”*

## Discussion

This study shows that the Filipinas in Norway face a number of challenges as immigrant women. Although they had different migratory pathways, the women had many shared experiences; worries about the future, homesickness, loneliness and concern for family members at home. The women were met with a number of political and economic stressors when trying to secure their future in Norway, such as obtaining suitable employment. The transnational roles that Filipinas occupy place additional pressure on them because, at times, they can struggle to meet the responsibilities they have in the Philippines and in Norway. Some mothers were also separated from their children, which had significant effects on their mental health. Immigration policy can contribute to and maintain power imbalances, resulting in the marginalisation of immigrant women [26]. This can impact not only mental health problems but also the resources women have to deal with them.

Language was commonly identified by the women as key to being part of Norwegian society. Language proficiency is also linked to access to, and knowledge of, health and social services [45]. According to Norwegian regulations, those who have residency based on having a spouse/partner in Norway have the right to, and are obligated to take part in, a minimum of 600 h of free Norwegian tuition (of which 50 h are social studies about Norwegian society) [46]. This has recently increased from 300 h, following an increased focus on acquiring language skills. However, until permission to stay is granted, the person is not entitled to this nor can he/she legally work. This can reinforce these women’s sense of social isolation, dependence on spouse/partner and feelings of low self-worth. Additionally, labour migrants are not entitled to this free tuition.

We also see that the very jobs which increase the demand for women’s migration serve to reinforce traditional gender roles [9] and hold women in socially disadvantaged positions. Au pairs for instance, have neither the rights of a student nor the protection of an employee [14], allowing for a large power differential between the privileged host family and the young, foreign au pair and thus, the potential for exploitation [16]. Exploitation and abuse are linked to poorer health among foreign migrant domestic workers [47].

Despite various immigration challenges, the informants showed a great deal of agency; they were resourceful, independent and adaptive. They worked hard to secure a future in Norway, not only in order to help their families at home but also to pursue their own hopes and dreams. The young, unmarried women felt particularly empowered through their migration, confirming previous research suggesting that women who migrate to support family also have their own agendas [48]. In contrast, the women who migrated through family reunification appeared to experience disempowerment and dependence, due to greater (initial) social and economic reliance on their spouse/partner. The women however, often sought advice from other Filipinas about education and job-hunting in order to improve their future earnings and increase their independence.

Overall, the women employed a number of coping strategies to deal with their loneliness, their guilt for being absent as a caregivers, their stress as breadwinners and other competing demands. Informal support from friends and family was utilised to a great extent, which falls in line with the importance Filipinos place on social relationships [33]. Informal sources of support have frequently been documented as preferable to formal sources among Asian immigrants, including Filipinos [49]. Being part of such a group, formal or informal, strengthens ethnic identification, which is associated with lower levels of depressive symptoms [44] and provides strong emotional, practical and social support. An Australian study that found that Filipina immigrants married to Australians sometimes severed family ties due to being unable to meet demands for remittances [19]. In contrast, a number of informants in our study still considered family the biggest source of support and they emphasised the importance of maintaining transnational ties.

The role of religion was also highlighted by our informants. Some reported increasing their church attendance. Previous research with Filipinos labour immigrants in Hong Kong found that church attendance promoted successful coping and reduced the impact of the emotional costs of migration [50]. This was due to the church groups being tailored towards immigrants. The women in the current study however, emphasised that it was their religious beliefs which gave them strength and guided them through difficult times. This is in line with previous research that indicates it is the spiritual side of religion which is associated with better mental health among Filipinos [51]. However, the same study found that spirituality is also associated with lower rates of professional help-seeking.

Recent research suggests that Filipinas are less likely to attend a GP for mental health problems, purchase psychotropic medication or engage in conversational therapy than Norwegian women are [20]. Our study suggests that Filipinas may often choose other ways to cope with their difficulties, since none had sought professional help despite five indicating that they were suffering from significant levels of psychological distress around the time of the interview. Some women may have benefited from professional help. Ways of improving professional help-seeking, as well as closer evaluation of the availability of alternative sources of help should therefore be explored in future research More specifically, studies investigating Filipina immigrants’ attitudes to seeking help for mental health problems in Norway and their experiences with the Norwegian health care system in general may help facilitate new ways of thinking in the provision of health and social services, as well as giving insight to the appropriateness of such services. Regardless, health care providers should be aware of the importance of considering the context of immigrant women’s lives in both their home country and host country when identifying and managing mental health problems among immigrants.

### Strengths and limitations

There are some important methodological considerations with regards to trustworthiness of the study [52]. To enhance the transparency of the findings, we have described all stages of data collection and analysis, including a clear description of the informants. This also helps to facilitate with transferability of the findings. The study highlights the importance of considering contextual factors in Filipina immigrants’ lives is relation to their mental health. While the study suggests that Filipinas have different and somewhat mixed experiences, there are some common elements that may be transferable not only to Filipinas in Norway but also to other immigrants who have transnational caregiving or breadwinning roles around the world. It should be noted though, that majority of the informants had higher education, which is associated with successful personal, social and economic adaption [53]. Women with fewer resources may face greater challenges.

With regards to credibility [52], recording and transcribing the interviews helped ensure the quality of the data. The first author, who interviewed the informants, is from the United Kingdom but lives in Norway. Coming from a different cultural background and having the role as a researcher, she may have been considered an outsider to the women. Yet, with some shared experiences of being an immigrant woman in Norway, the informants may have been more comfortable in discussing topics related to their difficulties in Norway. Findings are dependent on the subjective interpretations of the researchers. Although the first author had the main responsibility for data analysis, the data was discussed with the other authors, who also had immigrant backgrounds. Further, the second author is a Filipina living in Norway. The combination of backgrounds enabled an exploration of different perspectives, contributing further to the credibility of the findings. Attention was also given to contradictions in the data and instances of findings that did not apply to some informants have been reported.

## Conclusions

This study has explored how the social, political and cultural contexts of Filipina immigrants’ lives can impact mental health and coping through using the post-colonial feminist perspective. Structural factors that Filipinas face as immigrants can disempower them. Additionally, they occupy various transnational roles as women which can exacerbate stress and distress. Yet, Filipinas show agency and resilience that helps in their adjustment and they utilise social support networks and religion to help cope with their difficulties. Nonetheless, some women may not cope effectively and may benefit from professional help-seeking. Future research should consider attitudes to, and ways of improving, help-seeking from various sources.

## Abbreviations

GP: General practitioner
HSCL: Hopkins symptoms checklist

## References

[1] Bhugra D (2004). Migration and mental health. Acta Psychiatr Scand.

[2] Lindert J, Schouler-Ocak M, Heinz A, Priebe S (2008). Mental health, health care utilisation of migrants in Europe. Eur Psychiatry.

[3] Scheppers E, Van Dongen E, Dekker J, Geertzen J, Dekker J (2006). Potential barriers to the use of health services among ethnic minorities: a review. Fam Pract.

[4] Llácer A, Zunzunegui MV, del Amo J, Mazarrasa L, Bolůmar F (2007). The contribution of a gender perspective to the understanding of migrants’ health. J Epidemiol Community Health.

[5] Statistics Norway. Key figures for immigration and immigrants [Internet] 2017 [cited 2017, June 12]. Available from: https://www.ssb.no/en/innvandring-og-innvandrere/nokkeltall.

[6] Statistics Norway. Foreign citizens by citizenship and sex. 1st January [Internet]. ssb.no. 2016 [cited 2017 Jun 9]. Available from: https://www.ssb.no/256015/foreign-citizens-by-citizenship-and-sex.1-january.

[7] Asis MMB. The Philippines: Beyond Labor Migration, Toward Development and (Possibly) Return. Migration Information Source. July 12, 2017. http://www.migrationpolicy.org/article/philippines-beyond-labor-migration-toward-development-and-possibly-return.

[8] The Central Bank of the Philippines. Media Releases [Internet]. 2015. Available from: http://www.bsp.gov.ph/publications/media.asp?id=3664. [cited 2015 Jun 17].

[9] Tyner JA (1999). The Global Context of Gendered Labor Migration From the Philippines to the United States. Am Behav Sci.

[10] Ball RE (2004). Divergent development, racialised rights: globalised labour markets and the trade of nurses—The case of the Philippines. Womens Stud Int Forum.

[11] Sandnes T, Østby L. Family migration and marriage patterns 1990–2013 [Norwegian] [Internet]. Oslo: Statistics Norway; 2015 [cited 2015 Oct 12]. Report No.: 2015/23. Available from: http://www.ssb.no/befolkning/artikler-og-publikasjoner/familieinnvandring-og-ekteskapsmonster-1990-2013

[12] Yin J. The Economic Assimilation of South Asian Immigrants in Norway [Internet]. [Oslo]: University of Oslo; 2009 [cited 2015 Dec 10]. Available from: http://www.uio.no/forskning/tverrfak/culcom/publikasjoner/masteroppgaver/2009/pdf/JunYin-thesis.pdf

[13] Rosario TCD. Bridal Diaspora: Migration and Marriage among Filipino Women. Indian J Gend Stud 2005;12(2–3):253–73.

[14] Bikova M, Kontos M, Bonifacio G (2015). Au pair arrangement in Norway and transnational organization of care. Migrant Domestic Workers and Family Life: International Perspectives.

[15] Smaadahl T, Hernes H, Langberg L. Dreaming of the good life: A report on foreign national women, married to Norwegian men, who had to seek refuge in the shelters in 2001 [Internet]. Krisesenter sekretariatet: Oslo; 2002. Available from: http://www.krisesenter.com/backup240616/filer/pdf/materiell/Drommen-om-det-gode-liv-inenglish.pdf.

[16] Chuang JA. The U.S. Au Pair Program: Labor Exploitation and the Myth of Cultural Exchange. Harv J Law Gend 2013;36:269–343.

[17] de Castro AB, Gee GC, Takeuchi DT (2008). Workplace Discrimination and Health Among Filipinos in the United States. Am J Public Health.

[18] Thompson S, Hartel G, Manderson L, Woelz-Stirling N, Kelaher M (2002). The mental health status of Filipinas in Queensland. Aust N Z J Psychiatry..

[19] Thompson S, Manderson L, Woelz-Stirling N, Cahill A, Kelaher M (2002). The social and cultural context of the mental health of Filipinas in Queensland. Aust N Z J Psychiatry.

[20] Straiton ML, Powell K, Reneflot A, Diaz E. Managing Mental Health Problems Among Immigrant Women Attending Primary Health Care Services. Health Care Women Int 2016;37(0):1–22. 10.1080/07399332.2015.1077844 26251953

[21] Speziale HJS, Carpenter DR (2010). Qualitative research in nursing: Advancing the humanistic imperative. %th ed.

[22] Strand BH, Dalgard OS, Tambs K, Rognerud M (2003). Measuring the mental health status of the Norwegian population: A comparison of the instruments SCL-25, SCL-10, SCL-5 and MHI-5 (SF-36). Nord J Psychiatry.

[23] Benuto LT, Thalier NS, Leany BD. Guide to Psychological Assessment with Asians. Spring. 2014:479.

[24] Syed H, Zachrisson H, Dalgard O, Dalen I, Ahlberg N (2008). Concordance between Hopkins Symptom Checklist (HSCL-10) and Pakistan Anxiety and Depression Questionnaire (PADQ), in a rural self-motivated population in Pakistan. BMC Psychiatry.

[25] O’Mahony J, Donnelly T, Raffin Bouchal S, Este D (2013). Cultural Background and Socioeconomic Influence of Immigrant and Refugee Women Coping with Postpartum Depression. J Immigr Minor Health.

[26] O’Mahony JM, Donnelly TT (2010). A Postcolonial Feminist Perspective Inquiry into Immigrant Women’s Mental Health Care Experiences. Issues Ment Health Nurs.

[27] Anderson J, Perry J, Blue C, Browne A, Henderson A, Khan KB, et al. “Rewriting” Cultural Safety Within the Postcolonial and Postnational Feminist Project: Toward New Epistemologies of Healing. Adv Nurs Sci [Internet]. 2003;26(3). Available from: http://journals.lww.com/advancesinnursingscience/Fulltext/2003/07000/_Rewriting__Cultural_Safety_Within_the.5.aspx 10.1097/00012272-200307000-00005 12945655

[28] Hagelund A (2002). Problematizing culture: Discourses on integration in Norway. J Int Migr Integr Rev Integr Migr Int.

[29] Norwegian Directorate of Immigration. Want to apply: Skilled workers [Internet]. UDI. nd [cited 2016 Jul 1]. Available from: https://www.udi.no/en/want-to-apply/work-immigration/skilled-workers/?c=phl#link-816

[30] Seeberg ML, Sollund R, Shechory M, Ben-David S, Soen D (2010). Openings and Obstacles for Migrant Care Workers: Filipino Au Pairs and Nurses in Norway. Who pays the price? Foreign workers, society, crime and the law.

[31] Huang S, Yeoh BSA, Toyota M (2012). Caring for the elderly: the embodied labour of migrant care workers in Singapore. Glob Netw.

[32] Norwegian People's Aid. Au pair in Norway: A guide for au pairs and host families. Norwegian People's Aid: Oslo; 2015. Available from: file:///C:/Users/MELS/Downloads/HANDBOOK%20AUPAIR.pdf.

[33] Pe-Pua R, Protacio-Marcelino EA (2000). Sikolohiyang Pilipino (Filipino psychology): A legacy of Virgilio G. Enriquez Asian J Soc Psychol.

[34] Bikova M. In a minefield of transnational social relations: Filipino au pairs between moral obligations and personal ambitions. In: Cox R, editor. Au Pairs’ lives in global context: Sisters or servants? [Internet]. London: Palgrave Macmillan; 2014. p. 87–103. (Migration, Diasporas and Citizenship). Available from: https://books.google.no/books?id=hL8aBgAAQBAJ&pg=PA87&lpg=PA87&dq=In+a+Minefield+of+transnational+social+relations:+Filipino+Au+Pairs+between+Moral+Obligations+and+Personal+Ambitions&source=bl&ots=ve-FidVPGT&sig=H-hamsXa2_MyeoRxjpjMpgpFMiQ&hl=en&sa=X&ved=0CCIQ6AEwAWoVChMI-Yn7g-3xyAIVh8ByCh13zwRg#v=onepage&q=In%20a%20Minefield%20of%20transnational%20social%20relations%3A%20Filipino%20Au%20Pairs%20between%20Moral%20Obligations%20and%20Personal%20Ambitions&f=false

[35] Tacoli C (1999). International Migration and the Restructuring of Gender Asymmetries: Continuity and Change among Filipino Labor Migrants in Rome. Int Migr Rev.

[36] Perez LP. The Filipino Woman's Role in the Humanization of Social Life. In Wheatley, Christopher; Badillo, Robert; Calabretta, Rose; and Magliola Robert (editors). Humanisation of Social Life: Volume I: Theory and Challenges. Washington D.C.: Cultural heritage and contemporary change. Series VII. 2004.

[37] Basa C, De Guzman V, Marchetti S (2012). International migration and over-indebtedness: the case of Filipino workers in Italy [Internet].

[38] Mckay D (2007). “Sending Dollars Shows Feeling” – Emotions and Economies in Filipino Migration. Mobilities.

[39] Hochschild AR. Love and Gold. In: Hochschild AR, Ehrenreich B, editors. Global women: Nannies, maids and sex workers in the new economy [Internet]. New York: Owl Books; 2004 [cited 2015 Jun 15]. Available from: https://books.google.no/books?id=CBcrpIkb458C&pg=PA15&lpg=PA15&dq=love+and+gold+hochschild&source=bl&ots=XxAXRiMAdU&sig=8C4HBUqHw3l9twMrvjaY-p2I8Io&hl=en&sa=X&ved=0CBsQ6AEwADgKahUKEwjnmMTylvTIAhWJ3SwKHXELBVs#v=onepage&q=love%20and%20gold%20hochschild&f=false

[40] The Norwegian Directorate of Immigration. Permanent right of residence for family members of EU/EEA nationals [Internet]. N.D. [cited 2015 Oct 6]. Available from: http://www.udi.no/en/want-to-apply/permanent-residence/permanent-right-of-residence-for-family-members-of-eueea-nationals-/?e=y&c=phl

[41] Ko C (2012). Marital power relations and family life in transnational marriages: A study of Asian-French couples residing in France. EurAmerica J Eur Am Stud.

[42] Parreñas RS (2001). Mothering from a Distance: Emotions, Gender, and Intergenerational Relations in Filipino Transnational Families. Fem Stud.

[43] Ahmed A, Stewart DE, Teng L, Wahoush O, Gagnon AJ (2008). Experiences of immigrant new mothers with symptoms of depression. Arch Womens Ment Health.

[44] Mossakowski KN (2003). Coping with Perceived Discrimination: Does Ethnic Identity Protect Mental Health?. J Health Soc Behav.

[45] Lebrun LA (2012). Effects of length of stay and language proficiency on health care experiences among immigrants in Canada and the United States. Soc Sci Med.

[46] New in Norway. Tuition in Norwegian [Internet]. 2013 [cited 2016 Feb 9]. Available from: http://www.nyinorge.no/en/Familiegjenforening/New-in-Norway/Education-and-tuition-in-Norwegian/Tuition-in-Norwegian/Tuition-in-Norwegian-/

[47] Malhotra R, Arambepola C, Tarun S, de Silva V, Kishore J, Østbye T (2013). Health issues of female foreign domestic workers: a systematic review of the scientific and gray literature. Int J Occup Environ Health.

[48] Asis MMB (2002). From the Life Stories of Filipino Women: Personal and Family Agendas in Migration. Asian Pac Migr J.

[49] Yeh C, Wang Y-W (2000). Asian American coping attitudes, sources, and practices: Implications for indigenous counseling strategies. J Coll Stud Dev.

[50] Nakonz J, Shik AWY (2009). And all your problems are gone: religious coping strategies among Philippine migrant workers in Hong Kong. Ment Health Relig Cult.

[51] Abe-Kim J, Gong F, Takeuchi D (2004). Religiosity, spirituality, and help-seeking among Filipino Americans: Religious clergy or mental health professionals?. J Community Psychol.

[52] Graneheim U, Lundman B (2004). Qualitative content analysis in nursing research: concepts, procedures and measures to achieve trustworthiness. Nurse Educ Today.

[53] Berry JW (1997). Immigration, acculturation, and adaptation. Appl Psychol.

